# Phase I/IIa study to assess the safety, immunogenicity and efficacy of ChAdOx1-MVA vectored vaccines expressing a novel liver-stage malaria dual antigen LS2 by sporozoite challenge in malaria-naïve adults

**DOI:** 10.12688/wellcomeopenres.22900.1

**Published:** 2024-12-24

**Authors:** Daniel Silman, Amy Flaxman, Mehreen Datoo, Nick J. Edwards, Fernando Ramos-Lopez, Celia Mitton, Catherine Mair, Duncan Bellamy, Georgina Bowyer, Richard Morter, Benedict Halbroth, Navin Venkatraman, Pedro M. Folegatti, Julia Marshall, Ian Poulton, Amelia Bajer, Ahmed M. Salman, Eleanor Berrie, Jake Baum, Andrew M. Blagborough, Wendy Crocker, Rachel Roberts, Alison M. Lawrie, Alexandra J. Spencer, Sarah C. Gilbert, Katie J. Ewer, Adrian V. S. Hill

**Affiliations:** 1 Jenner Institute, Nuffield Department of Medicine, NIHR Biomedical Research Centre, University of Oxford Nuffield Department of Medicine, Oxford, England, OX3 7DQ, UK; 2 Centre for Clinical Vaccinology and Tropical Medicine, Churchill Hospital, Oxford, England, OX3 7LJ, UK; 3 Clinical Biomanufacturing Facility, University of Oxford, Churchill Hospital, Oxford, England, OX3 7JT, UK; 4 Department of Life Sciences, Imperial College London Faculty of Natural Sciences, London, England, SW7 2AZ, UK; 5 School of Biomedical Sciences, Faculty of Medicine and Health, University of New South Wales, Sydney, New South Wales, 2052, Australia; 6 University of Cambridge Department of Pathology, Cambridge, England, CB1 2QP, UK; 7 School of Biomedical Science and Pharmacy, The University of Newcastle College of Health Medicine and Wellbeing, Callaghan, New South Wales, 2308, Australia; 8 Global Health Vaccines R&D, GSK Vaccines Institute for Global Health, Siena, Italy

**Keywords:** P. falciparum; malaria; vaccines; CHMI; T cells; adenovirus; MVA; viral vectors

## Abstract

**Background:**

Induction of CD8 ^+^ T-cells using viral vectors is a promising strategy in developing effective vaccines against pre-erythrocytic malaria. A recent comparative assessment of candidate antigens using this approach in a mouse model had identified Liver Stage Antigen 1 (LSA1) and Liver Stage Associated Protein 2 (LSAP2) as more protective than TRAP and CSP antigens, which have been the dominant focus of clinical testing. We proposed that combining these within a novel dual antigenic insert (LS2), encoded alongside an orthologous immunogenic domain from invariant chain in ChAdOx1, and the F11 promoter in MVA, could translate to protective clinical efficacy against malaria.

**Methods:**

We conducted a non-randomised, open-label, dose escalation phase I/IIa study in UK adults, vaccinating a small lead-in group with ChAdOx1 LS2 5x10 ^9^ vp (group 1; n = 3) and subsequently a heterologous prime–boost group with ChAdOx1 LS2 2.5x10 ^10^ vp and MVA LS2 2x10 ^8^ pfu (group 2; n =10). Group 2 volunteers and 6 unvaccinated controls underwent Controlled Human Malaria Infection (CHMI) delivered by mosquito bite and standardized follow-up.

**Results:**

Vaccination with ChAdOx1 LS2 (both low and full doses) and MVA LS2 were generally well tolerated with solicited symptoms observed similar to previous vectored vaccines and no Severe Adverse Events (SAEs). Immunogenicity of the prime-boost schedule as measured by IFN-γ ELISpot was high showing median response of 4473 SFC/10^6 PBMC at the pertinent pre-challenge timepoint, covering a broad range of potential determinants. All vaccinated volunteers became infected with malaria during CHMI with a median time to diagnosis of 13 days compared to 13.25 days in controls.

**Conclusions:**

Though this study further indicates ChAd/MVA as a safe, highly effective platform for driving CD8 ^+^ responses specific to liver-stage malaria antigens, the promise of LSA1 and LSAP2 as potential candidates shown preclinically has not translated to protection from infection in humans.

**Clinical Trial Registration:**

ClinicalTrials.gov (Ref: NCT03203421), date of registration, 3 ^rd^ July 2017.

## Plain Language Summary

**A Phase I/IIa study to assess the safety, immunogenicity and efficacy of ChAdOx1-MVA vectored vaccines expressing a novel liver-stage malaria dual antigen LS2 in malaria-naive adults by sporozoite challenge**.

Malaria remains a significant public health issue causing hundreds of millions of cases and many thousands of deaths each year, mostly in young children living in sub-Saharan Africa. Although two vaccines have recently become available, they do not give total protection against malaria and the protection they give declines rapidly. New vaccines that give better protection that lasts longer would help further in controlling malaria.

Researchers developed a new vaccine targeting a different part of the malaria life cycle and tested this in UK adults that had never been exposed to malaria to test safety and measure the immune responses induced by the new vaccine. The study participants were also exposed to bites of mosquitoes infected with malaria to assess whether the vaccine protected against the disease.

Although the vaccine was shown to be safe and induced high levels of T cells, it did not protect volunteers from malaria and therefore further testing of the vaccine in its current form is not proposed.

## Introduction

Despite the recent prequalification and licensure of the first vaccines against malaria, current approaches in malaria vaccine development have so far fallen short of the high efficacy targets necessary to yield the required breakthroughs in malaria control ^1, 2^. Pivotal results from Phase III testing of the leading candidate RTS,S/AS01 _B_ highlighted the immense challenge of maintaining durable antibody-mediated protection against pre-erythrocytic antigens (circumsporozoite protein, CSP) ^3– 5^. Boosting at a later time-point only partially abrogates this ^4^. RTS,S/AS01 _B_ and another vaccine, R21/Matrix M are now recommended by the WHO and are being deployed in several countries however, the potential to achieve better efficacy and durability continues to fuel the search for better candidates.

Sporozoites migrate from the skin and infect hepatocytes in a short window ^6^, therefore the longer window (6.5 days at least) of liver-stage infection represents an attractive alternative or complementary vaccine strategy through the induction of CD8 ^+^ T cells capable of killing malaria-infected hepatocytes. Cellular immunity against liver stage antigens has been shown to have a role in protection against natural infection ^7– 9^ including severe disease and has been identified as the key determinant of protection against malaria challenge in both mice ^10– 13^, and non-human primates ^14^ immunised with irradiated sporozoites. Hepatocyte elimination by cytotoxic T lymphocytes (CTL) through MHC restricted recognition of parasite proteins on the hepatocyte surface is established as a likely effector mechanism ^15^. The use of viral vectors as a sub-unit vaccine platform to drive CD8 ^+^ T cell responses against liver stage malaria has undergone significant development in recent years, predominantly focusing on Thrombospondin Related Adhesion Protein (TRAP) ^16– 22^. TRAP fused to a multiple T cell epitope string (ME-TRAP) expressed in replication incompetent viruses ChAd63 (simian adenovirus) and MVA (modified vaccinia virus Ankara) administered in a heterologous prime-boost regimen resulted in 21% sterile protection with additional delay to patency in further volunteers in Phase IIa study. Efficacy appears to correlate with frequency of monofunctional IFN-γ producing CD8 ^+^ T cells ^16^.

Numerous steps have been taken in preclinical development to improve the efficacy of the viral-vectored liver stage malaria vaccines, within which identification of a more protective immunogen has been a key priority ^23^. Difficulties in pre-clinically assessing novel sub-unit candidates which lack an orthologue in standard rodent infecting *P. berghei* models has been recently circumvented with the generation of transgenic *P. berghei* parasites which express *P. falciparum* proteins ^24^. In a comparative assessment of a panel of eight candidate novel liver stage antigens carried out by Longley *et al.* at the University of Oxford, vaccination with ChAd/MVA in mice revealed Liver Stage Antigen 1 (LSA1) and Liver-Stage Associated Protein 2 (LSAP2) to be the most protective against transgenic *P. berghei* challenge, when tested separately in both inbred (LSA1 - 87.5% & LSAP2 87.5% sterile efficacy) and outbred mice (LSA1 - 87.5% & LSAP2 - 70% sterile efficacy). Both antigens were markedly superior to CSP and TRAP ^25^. Exact functions of these antigens are not fully elucidated, but both are known to be present within the parasitophorous vacuole (PV) during liver stage differentiation ^26, 27^ and neither antigen has received sustained focus in clinical trials. While LSA1 has been incorporated in both a protein-in-adjuvant formulation and a large, vectored polyprotein ^28, 29^, neither have been optimal for CD8 ^+^ T cell induction. HLA restricted CD8 ^+^ T cell responses to LSA1 epitopes have been associated with protection against severe infection in field studies ^7, 8^. Encoding these antigens as a combined insert—denoted as liver-stage dual antigen, LS2—within the same vector has potential to broaden the protective cellular response. Pre-clinical testing has shown that LS2 was more protective than single antigen vaccination, limiting the concerns of immune interference ^30^.

Further improvements to vaccine immunogenicity have been achieved preclinically by encoding the LS2 insert in ChAdOx1 alongside the short transmembrane domain of shark invariant chain ^31^ (Shark TM/Ii). Numerous vaccines incorporating intact human CD74 have been shown previously to enhance CD8 ^+^ T cell responses albeit through an unclear mechanism ^32– 38^ and it has been encouraging to replicate this approach preclinically using a safer (non-human) truncated orthologue. Attention has also been placed recently on the optimisation of the promoter sequence within the boosting MVA vector to enhance immunogenicity, potentially allowing for dose sparing and cost reduction. Our group in Oxford evaluating a candidate Middle East Respiratory Syndrome (MERS) vaccine has demonstrated that antigen expressed under the F11 promoter induces higher antigen specific cellular responses than both the standard p7.5 and mH5 promoters, but such an MVA vector has yet to be clinically tested ^39^.

The culmination of these developments resulted in the GMP production of new liver-stage viral vectored malaria vaccines ChAdOx1 LS2 (containing the LSA1-LSAP2 fusion protein linked to Shark TM/li) and MVA LS2 (expressing the LS2 fusion protein without Ii but expressed under the F11 promoter). In this study we assess the safety, immunogenicity, and efficacy of administration of these two vaccines in a standard heterologous regimen: prime with ChAdOx1 LS2 followed by boost with MVA LS2.

## Methods

### Study design and participants

We conducted an open-label, dose-escalation, first-in-human, phase I/IIa study in healthy malaria-naïve adult males and non-pregnant females between the ages of 18 and 45 years. Commencing in June 2017, recruitment and vaccination were conducted at the Centre for Clinical Vaccinology and Tropical Medicine at the University of Oxford. A lead-in group of three volunteers were the first to receive the new LS2 insert at one fifth of the established optimum dose for the ChAdOx1 vector. The sample size of the challenge group was chosen to reflect practical limitations on volunteer recruitment, ethical considerations limiting the number of volunteers that should receive a vaccine regimen without prior evidence of efficacy, and the desire to obtain preliminary description of safety, immunogenicity, and efficacy of the novel vaccines in a prime-boost regimen. Allocation to study group was undertaken by the investigators prior to enrolment based on subject preference: Group 1 (n=3) received a single vaccination low dose ChAdOx1 LS2 5 x 10 ^9^ virus particles (vp) and followed-up for 90 days. Group 2 (n=10) received full dose ChAdOx1 LS2 2.5 x 10 ^10^ vp followed by MVA LS2 2 x 10 ^8^ plaque forming units (pfu) 2 or 4 weeks later (a short prime-boost interval was selected based on recent findings from Ebola viral vectored vaccine trials which suggest no adverse impact of shortening the interval ^40^). Group 2 subjects underwent CHMI by mosquito bite at 3–4 weeks after final vaccination alongside unvaccinated infectivity control volunteers (control group A, n=6; included three control subjects from a concurrent CHMI study - ClinicalTrials.gov Identifier: NCT02905019).

### Ethics and consent

Potential volunteers were recruited by use of an advertisement and a registration form formally approved by the ethics committees (see below). All volunteers attended a screening visit, which took place up to 90 days prior to vaccination. The trial information sheet was made available to the volunteer at least 24 hours prior to the screening visit. Informed consent was taken before screening, as described in the trial protocol. If consent was obtained, the procedures indicated in the schedule of attendances was undertaken including a medical history, physical examination, blood tests and urine test with additionally having ECG if screening to participate in CHMI.

The study was conducted according to the principles of the Declaration of Helsinki and in accordance with Good Clinical Practice (GCP). The study was approved by the UK National Research Ethics Service, Committee South Central–Berkshire (Ref: 17/SC/0163) on the 4 ^th^ of May 2017, the Medicines and Healthcare Products Regulatory Agency (Ref: 21584/0374/001-0001) on the 26 ^th^ of May 2017, and the Oxford University Clinical Trials and Research Governance team, who independently and externally monitored compliance with Good Clinical Practice guidelines. Viral-vectored vaccine use was authorised by the Genetically Modified Organisms Safety Committee (GMSC) of the Oxford University Hospitals NHS Trust (Reference number GM 462.17.94). The trial was registered with ClinicalTrials.gov (Ref: NCT03203421) on the 3 ^rd^ of July 2017 (URL: Record History | ver. 2: 2017-07-03 | NCT03203421 | ClinicalTrials.gov) and an independent local safety monitor provided safety oversight.

### Vaccines

ChAdOx1 LS2 (Batch 02N16-01) was manufactured and vialed under Good Manufacturing Practice conditions at the Clinical Biomanufacturing Facility, University of Oxford. MVA LS2 (Lot: 0020517) was supplied by contract manufacturer IDT (Rosslau, Germany), who produced clinical lots under Good Manufacturing Practice. Both vaccines were stored at -70 to -85 ^⍛^C.

## Clinical procedures

### Inclusion criteria

This study was conducted in volunteers who met the following inclusion criteria: they were healthy adults between 18 and 45 years of age; able and willing (in the Investigator’s opinion) to comply with all study requirements; willing to allow the investigators to discuss the volunteer’s medical history with their General Practitioner; for females only, willing to practice continuous effective contraception during the study and a negative pregnancy test on the day(s) of screening and vaccination (see below); have agreed to refrain from blood donation during the course of the study and provided written informed consent to participate in the trial. There were various additional inclusion criteria for challenge volunteers (Group 2 and infectivity controls) which included agreement to refrain from blood donation during the study and for at least 3 years after the end of involvement in the study; being reachable (24/7) by phone during the period between CHMI and completion of antimalarial treatment; willingness to take a curative anti-malaria regimen following CHMI; for volunteers living out of Oxford: agreement to stay in a hotel near to the trial centre from at least day 6.5 post mosquito bite until antimalarial treatment is completed; and ability to answer all questions on the informed consent quiz correctly.

Effective and acceptable forms of contraceptive for female volunteers included established use of oral, injected or implanted hormonal methods of contraception. Volunteers using hormonal contraception must have used an effective additional and/or alternative method of contraception whilst on Riamet treatment, and until the start of the next menstruation after treatment. Further methods included placement of an intrauterine device (IUD) or intrauterine system (IUS); total abdominal hysterectomy; barrier methods of contraception (condom or occlusive cap with spermicide); male sterilisation, if the vasectomised partner was the sole male partner for the subject; and true abstinence, when in line with preferred and usual lifestyle of the subject [periodic abstinence such as calendar, ovulation, symptothermal, post-ovulation etc; a declaration of abstinence or the withdrawal method were not acceptable methods of contraception].

### Exclusion criteria

There were also extensive exclusion criteria. These included a history of clinical malaria (any species); travel to a clearly malaria endemic locality during the study period or within the preceding six months; receipt of an investigational product in the 30 days preceding enrolment, or planned receipt during the study period; prior receipt of an investigational vaccine likely to impact interpretation of the trial data as assessed by the investigator (including non-malaria adenovirus vectored experimental vaccines)--if any Group 2 underwent rechallenge, this criterion did not extend to vaccines previously received in this trial—; any confirmed or suspected immunosuppressive or immunodeficient state (including HIV infection, asplenia, recurrent severe infections and chronic immunosuppressant medication within the previous six months—inhaled or topical steroids are allowed); use of immunoglobulins or blood products within 3 months prior to enrolment; history of allergic disease or reactions likely to be exacerbated by any component of the vaccine (e.g. egg products, Kathon) or malaria infection; any history of anaphylaxis post vaccination; history of clinically significant dermatitis; pregnancy, lactation or intention to become pregnant during the study; history of cancer (except basal cell carcinoma of the skin and cervical carcinoma in situ); history of serious psychiatric condition that may affect participation in the study; any other serious chronic illness requiring hospital specialist supervision; suspected or known current alcohol abuse as defined by an alcohol intake of greater than 42 standard UK units per week; suspected or known injecting drug abuse in the 5 years preceding enrolment; hepatitis B surface antigen (HBsAg) detected in serum; seropositivity for hepatitis C virus (Abs to HCV) at screening (unless has taken part in prior hepatitis C vaccine study with confirmed negative HCV Abs prior to participation in that study, and negative HCV RNA PCR at screening for this study); inability of the study team to contact the volunteer’s GP to confirm medical history and safety to participate; any clinically significant abnormal finding on biochemistry or haematology blood tests, urinalysis or clinical examination; any other significant disease, disorder or finding that may significantly increase the risk to the volunteer because of participation in the study, affect the ability of the volunteer to participate in the study or impair interpretation of the study data.

There were also additional exclusion criteria for challenge volunteers (Group 2 and infectivity controls). These included clinically significant disturbances of electrolyte balance (e.g., hypokalaemia or hypomagnesaemia); use of systemic antibiotics with known antimalarial activity within 30 days of CHMI (e.g., trimethoprim-sulfamethoxazole, doxycycline, tetracycline, clindamycin, erythromycin, fluoroquinolones and azithromycin); history of sickle cell anaemia, sickle cell trait, thalassaemia, thalassaemia trait or any haematological condition that could affect susceptibility to malaria infection; use of medications known to cause prolongation of the QT interval ***and*** existing contraindication to the use of Malarone; use of medications known to have a potentially clinically significant interaction with Riamet ***and*** Malarone; contraindications to the use of ***both*** Riamet ***and*** Malarone; any clinical condition known to prolong the QT interval ***and*** existing contraindication to the use of Malarone; family history of congenital QT prolongation or sudden death ***and*** existing contraindication to the use of Malarone; history of cardiac arrythmia, including clinically relevant bradycardia ***and*** existing contraindication to the use of Malarone; positive family history in both 1 ^st^ and 2 ^nd^ degree relatives <50 years old for cardiac disease; inability for volunteer to be closely followed for social, geographical or psychological reason.

The following AEs associated with any vaccine constituted absolute contraindications to further administration of an IMP to the volunteer in question: anaphylactic reaction following administration of the vaccine; pregnancy. The following AEs constituted contraindications to administration of the vaccine at that point in time. If any one of these AEs occured at the time scheduled for vaccination, the subject may have been vaccinated later, or withdrawn, at the discretion of the investigator: acute disease (defined as the presence of moderate or severe illness with or without fever) at the time of vaccination; a temperature of ≥ 37.5°C (99.5°F) at the time of vaccination. All vaccines could be administered to persons with a minor illness such as diarrhoea, mild upper respiratory infection with or without low-grade febrile illness (I.e., temperature of ≥ 37.5°C (99.5°F)).

The following constituted absolute contraindications to CHMI: acute disease (defined as moderate or severe illness with or without fever); pregnancy.

### Vaccination

On the day of vaccination, vaccines were thawed to room temperature and administered intramuscularly into the deltoid of the non-dominant arm within 1 hour of removal from the freezer. Following vaccination, the injection site was covered with a sterile dressing and the volunteer stayed in the clinical area for 60 minutes (+15/-5 minutes) post-vaccination. Further observations were taken 30 minutes after vaccination (+/-5 minutes) and the sterile dressing was removed and injection site inspected. Volunteers were given a digital thermometer. Injection site reaction measurement tool and access to an electronic symptom diary to record their daily temperature, injection site reactions, solicited local and systemic adverse events for 7 days post-vaccination and unsolicited adverse events for 28 days post-vaccination. Volunteers attended regular vaccination follow-up visits–Group 1 volunteers up to 90 days post vaccination (on D2, D7, D14, D28, D56 & D90); Group 2 volunteers 90 days post CHMI (on D2, D7, D14 & D28 post ChadOx1 and on D2, D7 & D14 post MVA, as well as the day before CHMI—(C-1 is D76, C+90 is D174 post vaccination))–for review of clinical and laboratory adverse events (AEs) and blood tests. Local and systemic reactogenicity was evaluated at subsequent clinic visits and graded for severity, outcome and association to vaccination. AEs were assessed for relation to intervention and severity according to Tables 1, 2 and 3 in the extended data. Blood was sampled at all visits post-vaccination.

### CHMI

The volunteers were infected using five infectious bites from *P. falciparum* 3D7-strain infected *Anopheles stephensi* mosquitoes at Imperial College, London - supplied by the Department of Entomology, Walter Reed Army Institute of Research, Washington DC, USA. Post-CHMI volunteers were reviewed on D6 post-CHMI in the evening (C+6.5) and then twice a day, morning and evening between C+7 and C+14. Undiagnosed volunteers were reviewed once a day in the morning between C+15 and C+21. At each visit, blood was sampled for microscopy and qPCR. Physical observations were performed, and AEs were solicited. A diagnosis of blood stage malaria infection was made in subjects with symptoms suggestive of malaria and positive thick film microscopy, or qPCR result >500 parasites/ml if either thick film was negative or symptoms were absent ^22^ as summarised in Table 4 in the extended data. On diagnosis, volunteers were treated with a 3-day curative course of oral Riamet where every other dose was directly observed in clinic at time of diagnosis, 24- and 48-hours post-diagnosis. Volunteers intolerant of or having contraindications to Riamet were prescribed as appropriate alternative (oral Malarone or Chloroquine). Volunteers were reviewed 24- and 48-hours post-diagnosis where blood was sampled for microscopy. Provided these two blood films were negative for parasites, volunteers were not reviewed again in clinic until C+35. If one of these blood films were positive, volunteers continued to be reviewed in clinic at 24-hour intervals until two consecutive blood films were negative. Volunteers were then reviewed at C+35 and C+90 where safety assessments were conducted.

The blood tests were carried out as follows. Full blood count with differential, platelet count and serum biochemistry (including electrolytes, urea, creatinine, bilirubin, alanine aminotransferase, alkaline phosphatase and albumin) were measured at all visits before CHMI (except on Day 2 and Day 14 post each vaccination), at visit C+9, within 12 hours of diagnosis and at visit C+35. Blood was sampled for exploratory immunology studies at all visits before CHMI, C-1, C+7, C+35 & C+90.

### Outcomes

The primary outcome measures were to assess safety and tolerability of both ChAdOx1 LS2 and MVA LS2 and their efficacy (occurrence of blood stage malaria infection) in a heterologous prime-boost regimen against malaria sporozoite challenge, in healthy malaria-naïve volunteers. Secondary outcome measures were to assess immunogenicity and explore more sensitive efficacy outcomes including time to *P. falciparum* parasitaemia and parasite density dynamics (assessed by PCR) in healthy malaria-naïve volunteers.

### Immunological assays - ELISpot

The principal immunological readout in this trial was *ex vivo* Interferon γ (IFN-γ) Enzyme Linked Immunosorbent Spot (ELISpot) assay. This assay was performed using fresh peripheral blood mononuclear cells (PBMCs) to determine responses to LSA1 and LSAP2 longitudinally throughout the trial; from day of first vaccination until 90 days post infection. Assays were performed using Multiscreen IP ELISpot plates (Merck Millipore, Watford, UK) coated with 10 μg/mL human anti-IFN-γ antibody and developed using SA-ALP antibody conjugate kits (Mabtech, Stockholm, Sweden) and BCIP NBT-plus chromogenic substrate (Moss Inc., Pasadena, MA, USA). PBMCs were separated from whole blood with lithium heparin by density centrifugation within four hours of venepuncture. Cells were incubated for 18–20 hours in RPMI (Sigma) containing 1000 units/mL penicillin, 1 mg/mL streptomycin and 10% heat-inactivated, sterile-filtered foetal calf serum, previously screened for low reactivity (Labtech International, East Sussex, UK). 2.5 × 10 ^5^ PBMC were added to each well of the ELISpot plate in a final volume of 100 μL. Antigen consisted of pooled peptides, tested in triplicate, and the final concentration of each peptide per well was 10 μg/mL. Peptide sequences are described in Tables 5, 6 and 7 of the extended data.

These peptides spanned the complete LS2 vaccine insert, including Shark TM/Ii, LSA1, linker sequence and LSAP2; were each 15 amino acids in length; and overlapped by 10 amino acids. Pools for LSA1 and LSAP2 contained between 13 and 17 peptides. Staphylococcal enterotoxin B (0.02 μg/mL) and phytohaemagglutinin-L (10 μg/ mL) were pooled and used as a positive control. Plates were counted using an AID automated ELISpot counter (AID Diagnostika GmbH, algorithm C, Strassberg, Germany) using identical settings for all plates, and counts were adjusted only to remove artefacts.

Results are expressed as spot forming cells (SFC) per million PBMCs. This was calculated by subtracting the mean negative control response from the mean of each peptide pool response. Results are shown as either the summed response for the eight peptide pools or the responses for each of the individual pools. A quality control process was applied where plates were excluded if responses were >80 SFC/million PBMC in the negative control (PBMC without antigen) or <800 SFC/million PBMC in the positive control wells. One plate was excluded for failing QC (one assay at D0). Responses to the negative control were low, with a median of 4 SFC (interquartile range (IQR) 3–8). The lower limit of detection for the assay was 48 SFC for summed responses to all LS2 pools.

### Immunological assays – Epitope mapping

Epitope mapping of LSA1 and LSAP2 was performed using freshly separated PBMCs from Group 2 volunteers. This was done at a single timepoint; 7 days after CHMI. For each volunteer, peptide pools which had elicited high responses the day before CHMI (>500 SFC/million PBMC) were chosen. Individual peptides from these pools were tested in IFN-γ ELISpot assays as described above, except that the assay was performed in duplicate.

To investigate whether the Shark TM/Ii sequence contributed any T cell responses, the 4 individual peptides which made up the Shark TM/Ii peptide pool were tested in IFN-γ ELISpot assays as described above, except that frozen PBMCs were used, and the assay was performed in duplicate.

Quality control and expression of results for LSA1, LSAP2 and Shark TM/li mapping was performed as described above, as was expression of results.

### Immunological assays – Flow cytometry

Flow cytometry with intracellular cytokine staining (ICS) was performed at C-1. Samples for flow cytometry were stimulated in parallel with the *ex-vivo* ELISPOT using fresh PBMC. After overnight stimulation, samples were stained and acquired the same day on the Jenner Institute BD™ LSR II flow cytometer. Responses to LSA1 and LSAP2 were assessed using a single pool of peptides for each antigen. Details of staining and analysis are given in Table 8 of the extended data.

Responses were assessed using a 10-colour staining panel on freshly isolated PBMC, in parallel with ELISPOT assays. Aliquots of 2 × 10 ^6^ PBMC in 1 ml of media containing anti-CD28 and anti-CD49d at 1 μg/ml (eBioscience) and CD107a-PeCy5 (1:500, eBioscience) were stimulated with: no antigen; a pool of 56 peptides spanning the T9/96 strain of the TRAP antigen (20mers overlapping by 10 amino acids, at 2 μg ml−1); a pool of 56 peptides spanning the 3D7 strain of the TRAP antigen (20mers overlapping by 10 amino acids, at 2 μg ml−1); a pool of 31 peptides spanning the CS antigen (15mers overlapping by 11 amino acids, at 2 μg ml−1) or a positive control; Staphylococcal enterotoxin B (Sigma, 1 μg ml−1) in 5 ml polystyrene FACS tubes for 18 hours at 37°C and 5% CO _2_. Brefeldin A and Monensin, both at 1 μg/ml, were added for the last 16 h. Cells were incubated with a dead cell discrimination dye (AQUA 1:200, Invitrogen) for 20 minutes at room temperature. PBMCs were permeabilised, then stained intracellularly at room temperature for 30 minutes with CD4-APC (1:25, eBioscience) CD14- Pacific Blue and CD19-Pacific Blue (both 1:50, eBioscience), CD3-Alexa Fluor 700 (1:50, eBioscience), CD8-APC-Alexa Fluor 780 (1:10, eBioscience) and IFN-γ-FITC (1:100, eBioscience), IL-2-PE (1:50., eBioscience) and TNFα-Pe-Cy7 (1:500, eBioscience), then washed and fixed in 1% paraformaldehyde. Further details of monoclonal antibodies are given in Table 8 of the extended data. Compensation was performed using single-stained One-Comp beads (eBioscience) for monoclonal antibodies and ARC beads for AQUA (Life Technologies).

**Figure 1.**
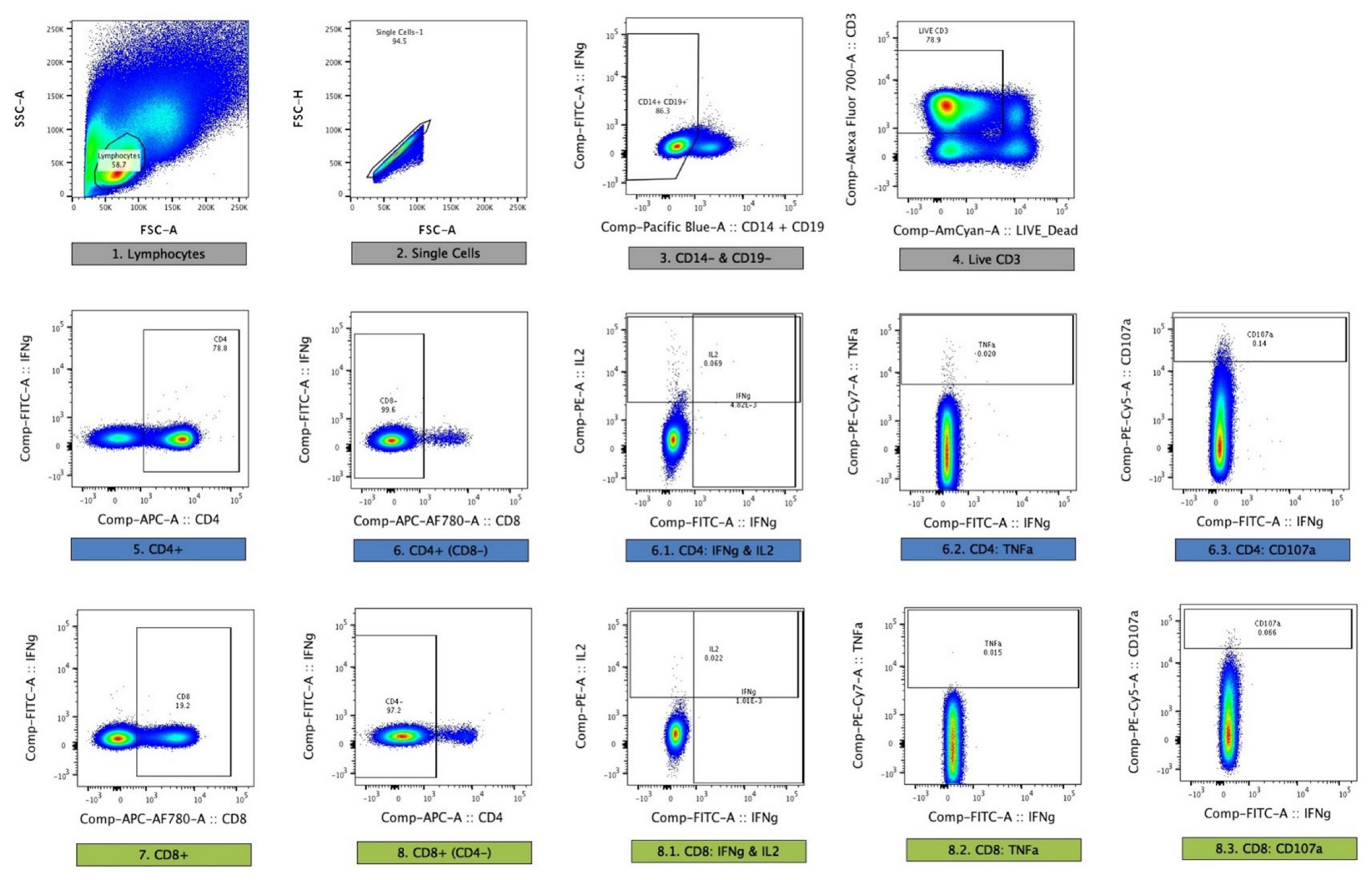
Gating strategy for flow cytometry. Singlets were identified using forward scatter plots. Dead cells were excluded by violet, fluorescent amine-reactive dye staining. Monocytes and B cells were excluded by CD14 or CD19 expression and T cells identified by CD3 expression. T cells were then subdivided by gating on CD4+ and CD8+ populations. Cytokine expression was quantified by plotting pairs of cytokines against each other and gating positive populations. This is a representative sample from a sample stimulated overnight (18 hours) with a single pool of overlapping LSA1 peptides.

Acquisition was performed on the day of staining on a BD LSRII with median of 501,000 live CD3 ^+^ cells acquired (IQR 25% = 361750, 75% = 627000) per sample. Data was prepared and analysis performed using FlowJo v9.6.2 (Treestar Inc). A hierarchical gating strategy was used ( Figure 1). Cells were gated on lymphocytes, singlets, live CD3 ^+^, CD8 ^-^CD4 ^+^ or CD4 ^-^CD8 ^+^ and then IFNγ, IL-2, TNFα and CD107a. Dead cells (AQUA+), monocytes (CD14 ^+^) and B cells (CD19 ^+^) were excluded from the analysis. All SEB stimulated PBMCs gave a cytokine response >1%. Responses to peptide were determined after subtraction of the response in the unstimulated control for each sample, and considered positive if the count was >20; the frequency higher than the autologous unstimulated control; and the lower limit of detection (LLD CD4 ^+^ = 0.0029, LLD CD8 ^+ ^= 0.0054). 

### Parasite quantitative Polymerase Chain Reaction (qPCR)

Vaccine efficacy measured as time to parasitaemia assessed by qPCR for *P. falciparum* was a secondary objective of the clinical trial and was conducted as described previously ^19^ with the following modifications. Blood was collected at baseline and at clinical protocol-defined time points following CHMI for qPCR in 2.0ml tubes containing EDTA. DNA was extracted from 0.4mL EDTA whole blood using a QiaSymphony SP robot, utilising the Qiagen DSP Blood Midi Kit and the pre-loaded Blood 400 v6 extraction protocol, with a 100μl elution in ATE buffer selected. 5μL of each extraction was used per assay well and was run in triplicate for qPCR (equivalent to 60μL blood directly assessed). Parasites per mL (p/mL) equivalent mean values were generated by a standard TaqMan absolute quantitation, against a defined standard curve of diluted *P. falciparum* 3D7 DNA, qualified against DNA from counted parasites in whole blood (previously extracted by the same method). qPCR was conducted on an ABI StepOne Plus machine with v2.3 software, using default Universal qPCR and QC settings, apart from the use of 45 cycles and 25μL reaction volume. This process has been formally validated as suitable for diagnostic purposes and qPCR detection is regularly externally assessed by participation in the UKNEQAS Malaria (Molecular) scheme.

### Statistical analysis

Data were analysed using GraphPad Prism version 7.03 or 8.1 for Windows (GraphPad Software Inc., California, USA) and Stata 10.0 (Statacorp LP, Texas, USA). All functions can be performed using R and R Studio. A chi-squared test or the Fisher’s Exact test was used to compare the safety data between different groups. For immunological read-outs, Kruskal-Wallis analysis with Dunn’s multiple comparisons test were used to compare responses post vaccination with baseline responses. Significance testing between two groups used Mann-Whitney analysis. A Wilcoxon matched-pairs analysis was used to compare between time points within groups. A statistically significant difference in efficacy between a vaccination regimen and controls was assessed by both Fisher’s Exact test in comparison of proportions protected and log rank analysis of Kaplan Meier curves at the different endpoints. The unpaired t test was used to compare log transformed parasite density values between groups. A value of p < 0.05 was considered significant.

## Results

### Participant flow

**Figure 2.**
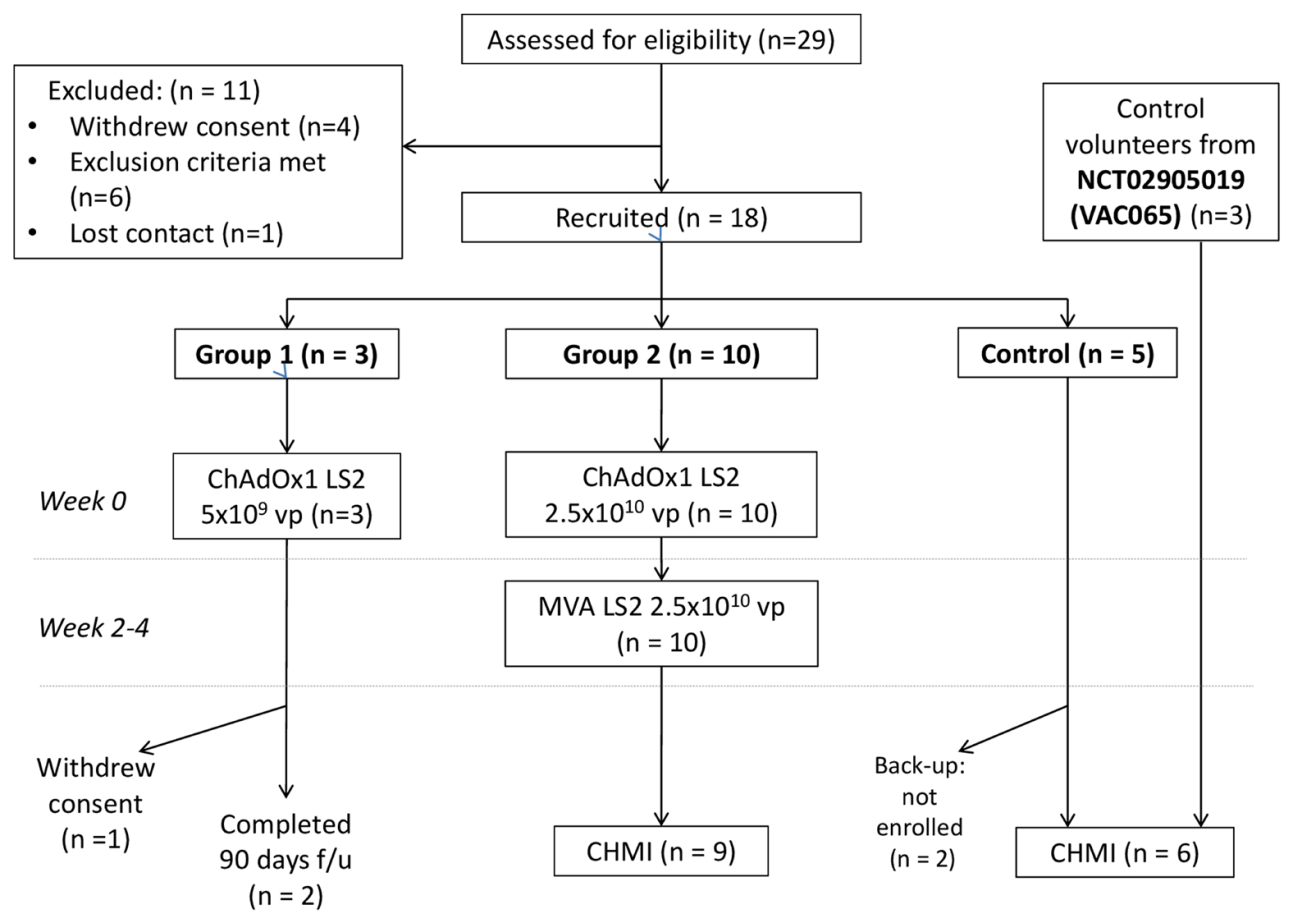
CONSORT diagram of participant activity during VAC067 clinical study ClinicalTrials.gov Ref: NCT03203421: Numbers screened, enrolled, vaccinated with ChAdOx1 LS2 and MVA LS2 (vp = viral particles; pfu = plaque forming units), undergoing controlled human malaria infection (CHMI) and withdrawn indicated as shown.

From June to September 2017, 29 individuals were screened for their eligibility of whom 11 were excluded or withdrew consent prior to enrolment. Three volunteers were allocated to Group 1 with one withdrawing after 28 days of follow-up and hence not replaced. All ten volunteers in Group 2 received both ChAdOx1 LS2 and MVA LS2 with one withdrawing their consent to undergo CHMI after vaccination. Neither withdrawal was related to safety concerns about vaccination. 3 volunteers plus a further 3 screened to a concurrent trial (Ref: NCT02905019) (control group A, n=6) completed CHMI and 90 days follow-up alongside the Group 2 volunteers. Two further eligible volunteers served as back-up controls though were not enrolled. Participant enrolment is summarized in Figure 2. Baseline demographic data is summarised in Table 1.

**Table 1.** Baseline data for participants in each group.

	Group 1 (n=3)		Group 2 (n=10)		Control (n=6)		Total (n=19)	
Sex								
Male	2	66.7%	5	50.0%	4	66.7%	11	57.9%
Female	1	33.3%	5	50.0%	2	33.3%	8	42.1%
Median Age	38		27		26		27	
Ethnicity								
White British	3	100.0%	5	50.0%	4	66.7%	12	63.2%
Other White	0	0.0%	4	40.0%	1	16.7%	5	26.3%
Other Asian	0	0.0%	0	0.0%	1	16.7%	1	5.3%
Other Background	0	0.0%	1	10.0%	0	0.0%	1	5.3%

### Adverse Events

**Figure 3.**
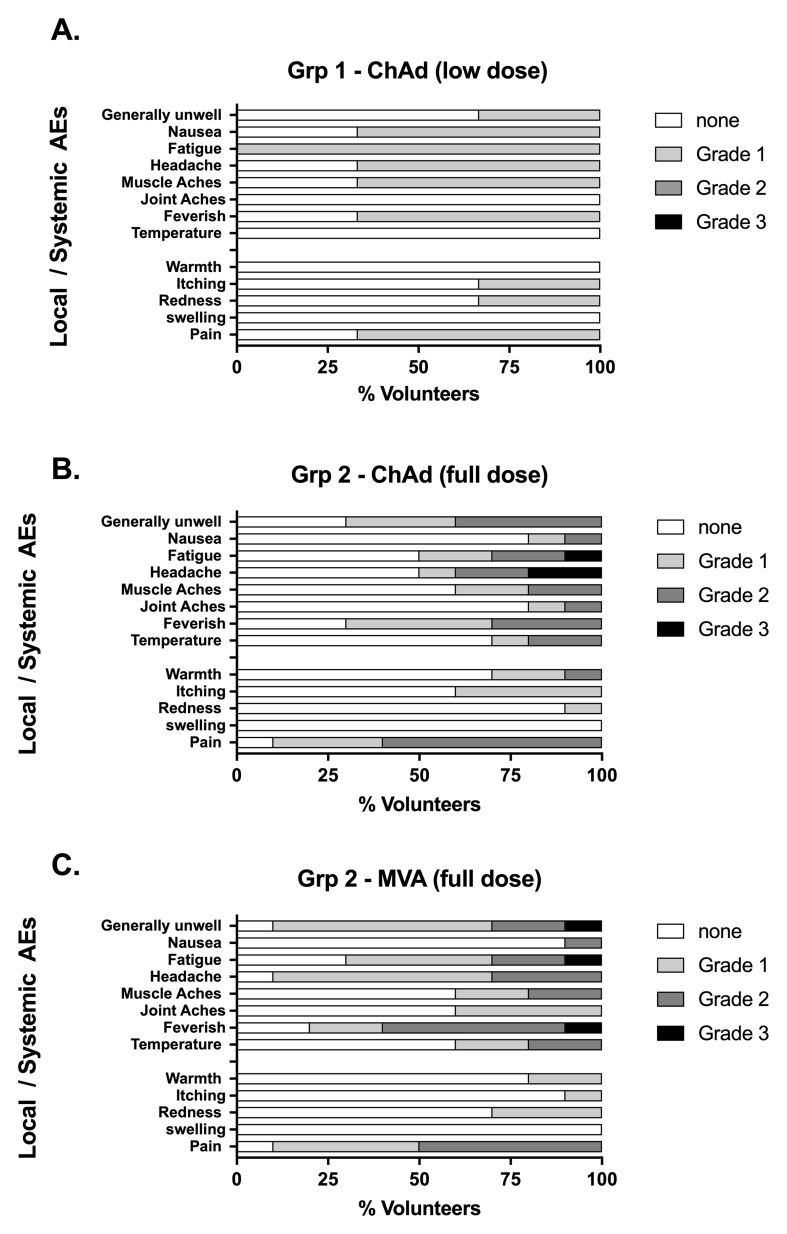
Safety data – occurrence of local and systemic solicited adverse events (AEs): Vaccinated participants recorded the occurrence of solicited for 7 days following vaccination in designated diaries, graded according to severity: 1 = mild, 2 = moderate, 3 = severe. Low dose = 5 x 10 ^9^ vp ( **A**), full dose = 2.5 x 10 ^10^ vp ( **B**); MVA full dose = MVA LS2 2 x 10 ^8^ pfu ( **C**).

All AEs following low dose vaccination were mild in severity ( Figure 3A). Local and systemic symptoms following vaccination with both full dose ChAdOx1 LS2 and MVA LS2 were relatively common (mean number AEs per participant were 5.2 and 6.0 respectively). Injection site pain, feverishness and general malaise were observed following most vaccinations with both vectors at the full dose ( Figures 3B & 3C). Most AEs were mild-to-moderate in severity. Pain was the most persistent AE, lasting a median of 3 days for both ChAdOx1 LS2 (range 1–5) and MVA LS2 (range 1–4) with most other AEs (90.4% for ChAdOx1 LS2 and 93.3% for MVA LS2) resolving within 48 hours. There were no SAEs, and unsolicited AEs assigned at least possible causation with vaccination were sporadic and generally mild in severity, as were changes to safety blood tests (Tables 9 and 10 of the extended data).

Overall, both ChAdOx1 LS2 and MVA LS2 were vaccinated within expected safety and tolerability limits. Comparison of ChAdOx1 LS2 against prior use of this novel vector in a candidate flu vaccine (ChAdOx1 NP +M1 Ref: NCT01818362 ^41^) revealed no statistical differences in frequency of AEs. The total occurrence of AEs (p <0.0015 Chi-squared test) did however appear to be significantly higher for ChAdOx1 LS2 than the current leading adenoviral vectored malaria candidate ChAd63 ME-TRAP (Ref: NCT01623557) including all moderate-severe AEs (p <0.0001 Fisher’s Exact test). Occurrence of key symptoms pain and fever were not significantly different to those vaccines. Breakdown of solicited AEs by severity and comparison with relevant vectors is summarised in Table 2. Vaccines were compared by Chi squared test (>) or Fisher’s Exact test (@, severity gradings grouped together to meet requirements for test: none/mild vs moderate/severe for pain, and none vs any grade for fever).

**Table 2.** All solicited AEs following vaccination with ChAdOx1 LS2 and MVA LS2 in this study (£ = NCT03203421). Shown with data from recent trials of viral vectored vaccines at University of Oxford ($ = 19 and % = 41). Vaccines were compared by Chi squared test (>) or Fisher’s Exact test (@, severity gradings grouped together to meet requirements for test: none/mild vs moderate/severe for pain, and none vs any grade for fever).

Vaccine	ChAdOx1 LS2	ChAd63 ME-TRAP	ChAdOx1 NP +M1	MVA LS2	MVA ME-TRAP	MVA NP +M1
* (study / identifier)*	*£*	*$*	*%*	*£*	*$*	*%*
**Dose Vaccine**	2.5x10 ^10^ vp	5x10 ^10^ vp	2.5x10 ^10^ vp	2x10 ^8^ pfu	2x10 ^8^ pfu	2x10 ^8^ pfu
**N enrolled**	10	15	24	10	15	12
**No. AEs by grade:**						
***None (pain [P]; fever [F])***	*78 (1; 7)*	*142 (7; 13)*	*189 (2; 21)*	*69 (1; 6)*	*80 (1; 9)*	*87 (0; 11)*
**Mild (P; F)**	25 (3; 1)	48 (7; 2)	73 (13; 3)	36 (4; 2)	71 (3; 4)	41 (5; 1)
**Moderate (P; F)**	24 (6; 2)	5 (1; 0)	40 (9; 0)	22 (5; 2)	32 (9: 2)	20 (5; 0)
**Severe (P; F)**	3 (0; 0)	0 (0; 0)	10 (0; 0)	3 (0; 0)	12 (2: 0)	8 (2; 0)
		**comparison to** ** ChAdOx1 LS2**			**comparison to MVA** ** LS2**	
*All Aes ^>^*		*0.0015	*0.3929*		*0.0791*	*0.4888*
*Moderate & Severe AEs ^@^*		*<0.0001	*0.2707*		*0.4923*	*0.8787*
*Severe AEs ^@^*		*0.0631*	*0.7637*		*0.1753*	*0.3552*
*pain ^@^*		*0.1782*	*0.2764*		*0.3973*	*>0.9999*
*fever ^@^*		*0.3577*	*0.3284*		*>0.9999*	*0.1353*

### Immunogenicity assessed by IFN-γ ELISpot data

**Figure 4.**
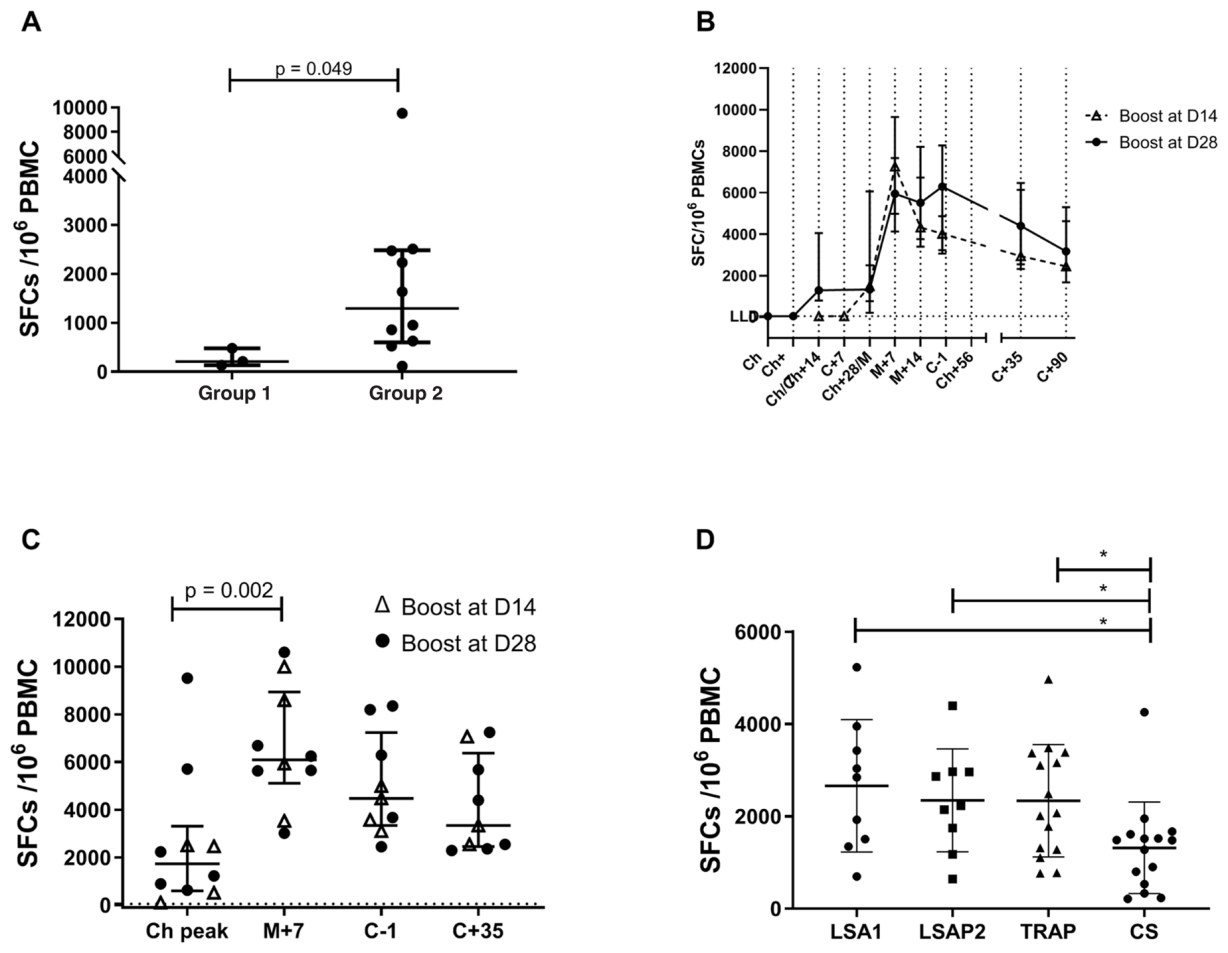
Summed LSA1/LSAP2 T cell responses to vaccination measured by IFN-γ ELISPOT. ( **A**) ELISPOT responses following ChAdOx1 LS2 administration after 14 days which is the median peak timepoint following prime, responses in group 1 (lead-in) and group 2. *p=0.049 by Mann-Whitney U comparison. Kinetics of responses in Group 2 volunteers ( **B**) and ( **C**), both showing median with IQR. Responses denoted with Δ (n=4) are volunteers vaccinated on shorter 2 week prime-boost interval for comparison against 4-week interval (denoted with ●, n = 6) In ( **B**) kinetics of response are shown such that day of boost and day before challenge for the two prime-boost intervals align, with different baseline timepoints. In ( **C**) individual data points are shown at key timepoints. Comparison using Kruskall-Wallis with significance between timepoints shown for Dunn’s Multiple comparison test. ( **D**) Magnitude of responses on day before challenge compared against viral vectored antigens ME-TRAP and CSP administered in NCT01623557. Significance bars * indicate Mann Whitney U comparison.

The magnitude and kinetics of T cell responses specific to LS2 peptides was assessed by IFN-γ ELISpot, by stimulating PBMCs with peptide sequences spanning both LSA1 and LSAP2. Responses were detectable in all volunteers after initial vaccination with ChAdOx1 LS2 and were higher in Group 2 than Group 1 ( Figure 4A). This reached significance at 14 days following ChAdOx1 prime which is the median peak (post-prime) timepoint (p= 0.049 by Mann Witney U comparison).

The time-course of summed median ELISpot responses for Group 2 participants is shown in Figure 4B, revealing classic T-cell response kinetics for viral vector administration ^16, 42– 45^. The Group 2 cohort included four volunteers (out of a group size of 10) receiving vaccination on a two-week prime-boost interval (14–15 days; denoted with Δ in Figure 4B and C) and six volunteers on the 4-week (23 – 28 days) interval (denoted with ● in Figure 4B and C). Comparison of the different prime-boost intervals is shown in Figure 4B. Post-boost ELISpot responses were not significantly different between the two different prime-boost intervals (p = 0.76 at M+7, p = 0.41 at C-1 respectively; Mann Witney U test). As a result, for further comparisons, data for all volunteers in Group 2, regardless of prime-boost interval are pooled together.

Individual ELISpot responses for Group 2 volunteers at key timepoints during the trial are shown in Figure 4C. The peak response after ChAdOx1 prime (14–28 days, 1731 SFC/10^6 PBMC) was significantly higher than the ELISpot responses seen at baseline (p = 0.002, Wilcoxon matched pairs signed rank test). Boosting with MVA LS2 significantly increased immunogenicity (compared to post ChAdOx1 peak) to a median of 6092 SFC/10^6 PBMC at 7 days post MVA (Dunn’s multiple comparison test, p = 0.002). Responses remained relatively preserved at C-1, (3 – 4 weeks post MVA), with a median response of 4473 SFC/10^6 PBMC ( Figure 4C). Follow-up of all Group 2 volunteers until 90 days post CHMI revealed small decline of ELISpot responses as anticipated ( Figure 4B). ELISpot responses to LS2 among infectivity controls were minimal when enrolled at C-1 and did not significantly expand during challenge follow-up (data not shown).

T-cell responses as measured by ELISpot to the constituent vaccine antigens LSA1 and LSAP-2 amongst Group 2 participants at C-1 were at similar levels to those seen against TRAP when vaccinated in viral vectors ChAd63/MVA in a previous study at the University of Oxford ^19^ (n= 15; Figure 4D, p=0.64 and p= 0.91 LSA1 and LSAP2 respectively versus TRAP, Mann Whitney test). Responses to LSA1 and LSAP-2 were higher than CSP, also expressed in ChAd63/MVA in the same study (n = 14; p=0.029 and p = 0.015 respectively; Mann Whitney test).

### Epitope mapping by IFN-γ ELISpot

**Figure 5.**
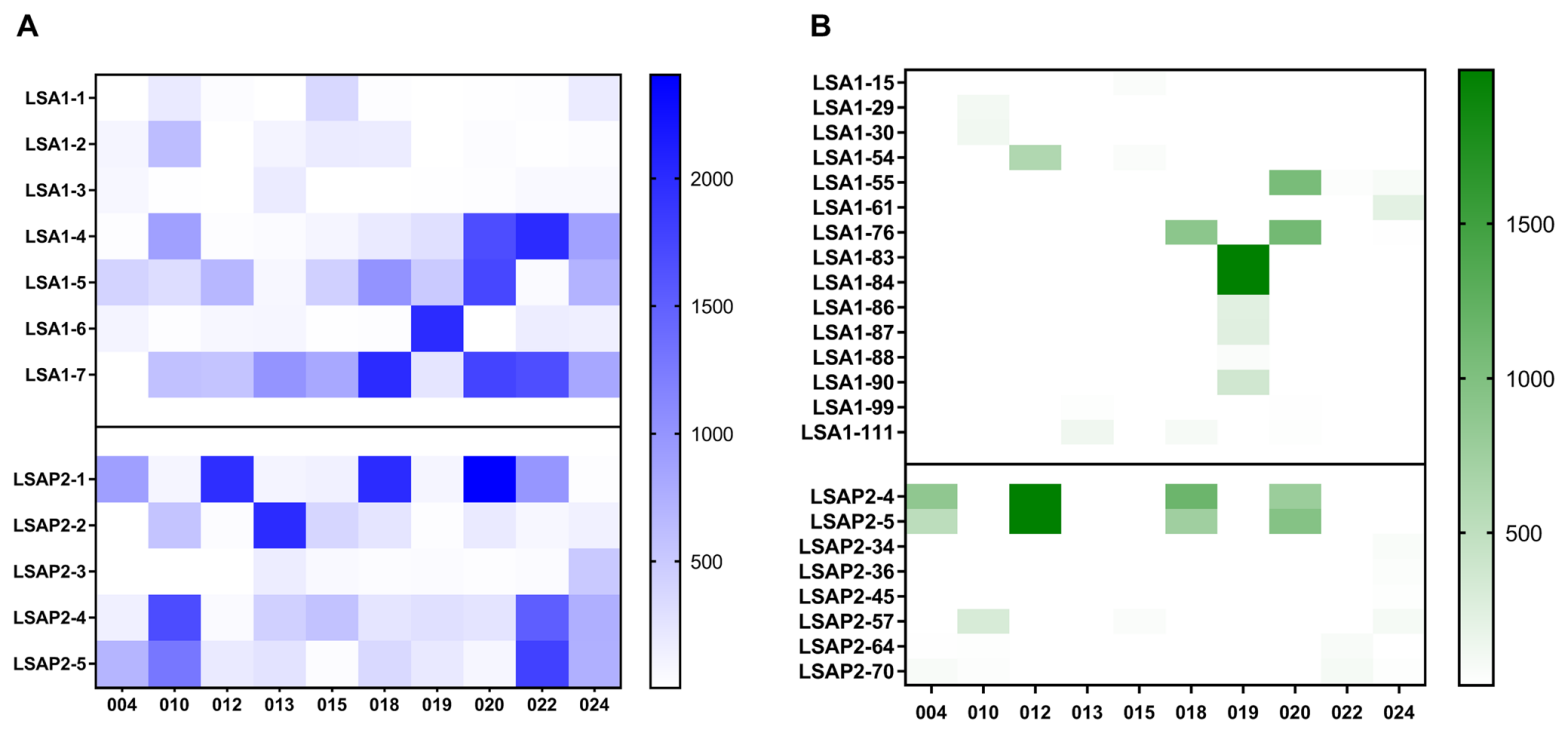
Heatmaps to show ELISpot responses to individual peptide pools ( **A**) Responses to each of the seven LSA1 and five LSAP2 peptide pools in group 2 volunteers on day before challenge. ( **B**) Responses to individual peptides in group 2 volunteers 7 days after challenge. Individual peptides for testing were selected from the pools with high responses in ( **A**). Only responses >10 SFC/million are shown.

To further investigate immunogenicity of LS2 using the ELISPOT assay, we assessed the responses to individual peptide pools. Across the 10 volunteers in Group 2, responses of greater than 500 SFC/million on the day before CHMI were elicited in 19 peptide pool/volunteer combination, with a range of 1 to 4 pools per volunteer ( Figure 5A). No single pool consistently elicited maximal response, mean responses to each pool ranging from 45 (LSA1–3) to 986 (LSA1–7) SFC /10^6 PBMC. Fine epitope mapping of these pools was carried out by IFN-γ ELISpot to determine which peptides in each pool were dominating the response ( Figure 5B). This was performed at D7 after CHMI in volunteers in Group 2. Most strikingly, we saw a response to LSAP2 peptides 4 and 5 of >500 SFC/million PBMC in 4 out of 10 volunteers.

**Figure 6.**
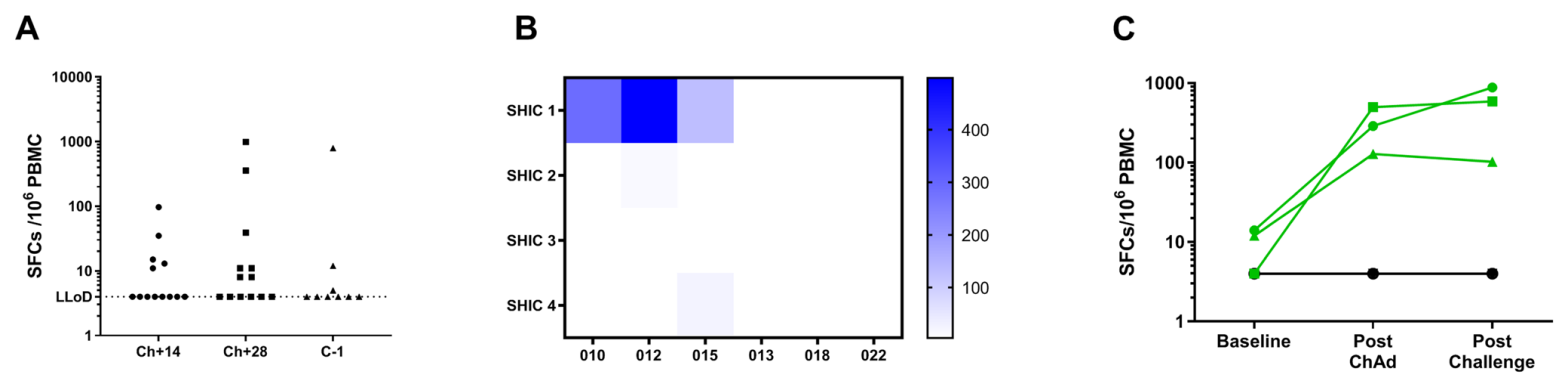
T cell responses to Shark invariant chain measured by IFN-γ ELISPOT. ( **A**) Individual responses to peptide pool (4 peptides) covering Shark TM/Ii fragment within the ChAdOx1 LS2 antigenic insert. Data is shown for all individuals in Group 1 and 2, at 3 key timepoints within the trial. ( **B**) Heatmap to show responses to each of the 4 peptides making up the Shark TM/Ii fragment in 6 volunteers before challenge – 3 of whom responded to the pool (010, 012, 015) and 3 who did not (013, 018, 022). ( **C**) Responses to SHIC1 peptide at 3 timepoints in the same 6 volunteers as in ( **B**). In ( **A**) assay was performed using fresh PBMCs, in ( **B**) and ( **C**) assay was performed using PBMCs from frozen.

We also measured T cell responses to the molecular adjuvant, Shark TM/Ii fragment ( Figure 6). In some volunteers, IFN-γ ELISpot responses in the range of 100 to 1000 SFC/million PBMCs were observed at multiple timepoints post vaccination. In other volunteers, responses to Shark TM/Ii remained around the lower limit of detection ( Figure 6A). Further investigation of this unexpected signal was performed, as Shark TM/Ii is included in the vaccine as a molecular adjuvant and was not expected to elicit T cell responses. Three responders and three non-responders to Shark TM/Ii peptide pool were selected. Responses to the four individual peptides were measured in these six volunteers, which showed that the response seen mapped to SHIC 1 peptide ( Figure 6B). This response was not present before vaccination and was only present in the responders after vaccination ( Figure 6C). Responses to five overlapping peptides spanning the human invariant chain sequence were also measured in these six individuals. Both before and after vaccination, responses in all six volunteers were negative. Since some volunteers respond to SHIC 1 peptide and some do not, we investigated whether this response was HLA restricted. Using NetMHC, we found that two peptide sequences within the Shark TM/Ii fragment (SLLWGGVTV and LLWGGVTVL, both of which fall within the SHIC1 peptide sequence MSLLWGGVTVLAAML) were predicted as strong binders in HLA*A02 individuals. All three responder volunteers tested were confirmed to be HLA*A02 positive and all three non-responders were confirmed to be HLA*A02 negative. To confirm this observation, we assessed responses to SHIC1 in the remaining volunteers in Groups 1 and 2. Eight volunteers were HLA*A02 negative and did not respond to SHIC1 at baseline or post vaccination. Six volunteers were HLA*A02 positive. None responded to SHIC1 at baseline, and 5 out of 6 showed responses to SHIC1 post vaccination (>50 SFC/million PBMCs).

### Immunogenicity assessed by flow cytometry

**Figure 7.**
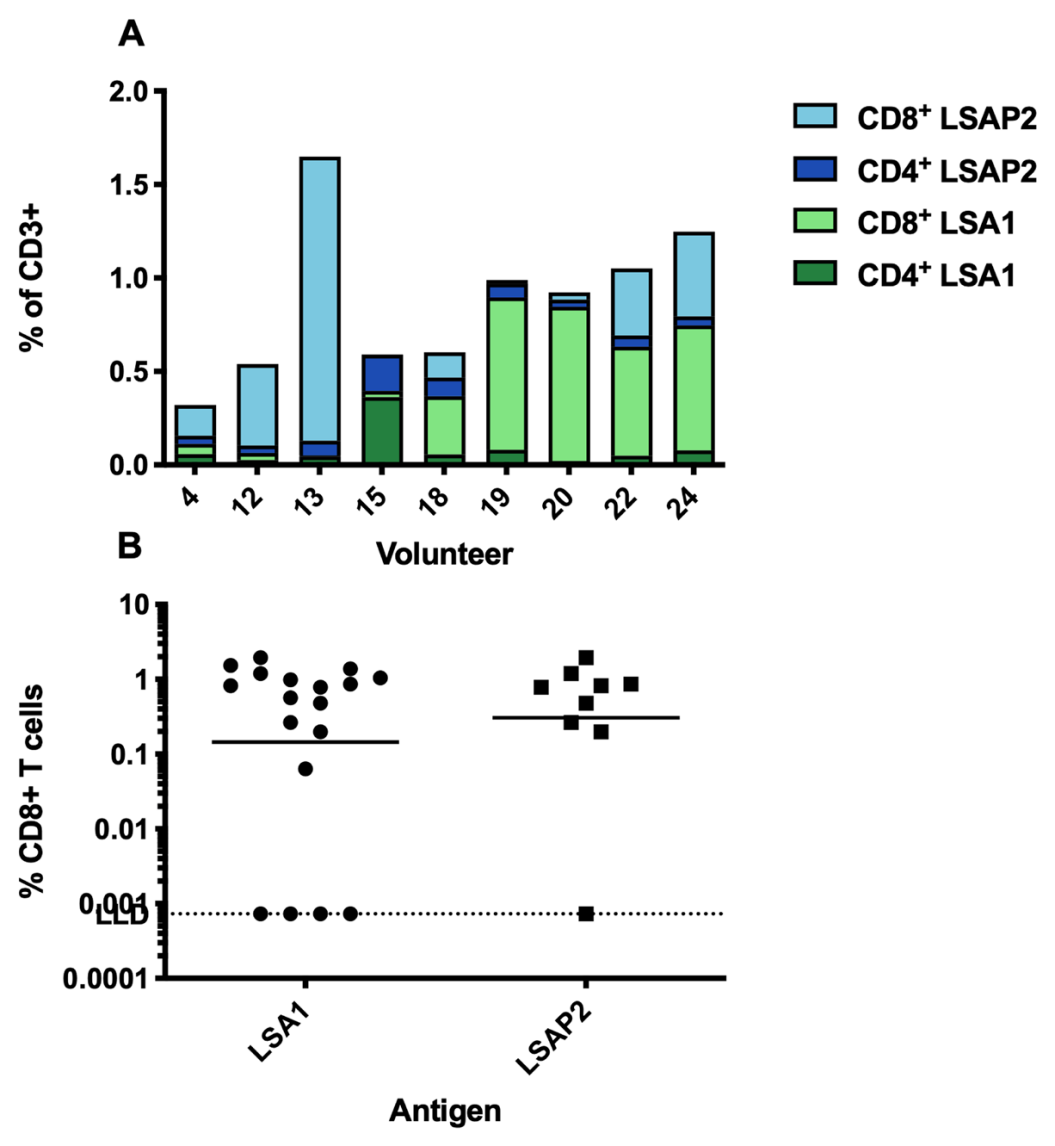
T cell responses measured by flow cytometry. ( **A**) Frequency of CD4 ^+ ^or CD8 ^+^ T cells expressing IFN-γ, IL-2 or TNFα recognising either LSA1 or LSAP2. ( **B**) Frequency of CD8 ^+^ T cells expressing CD107a ^+^.

Cytokine secretion was measured by flow cytometry with intracellular cytokine staining to determine the frequency of CD4 ^+^ or CD8 ^+ ^T cells expressing any cytokine (IFN- γ, IL-2 or TNFα) in response to LSA1 and LSAP2 peptide pools ( Figure 7A). The proportion of responses to each antigen varied substantially between participants, likely due to the recognition of different epitopes through different HLA alleles. Eight of the nine participants showed responses that were dominated by CD8 ^+^ T cells specific for either LSAP2 or LSA1. Antigen-specific CD8 ^+^ T cells demonstrated cytotoxic capacity by expression of the degranulation marker CD107a in most participants ( Figure 7B).

### Vaccine efficacy

**Figure 8.**
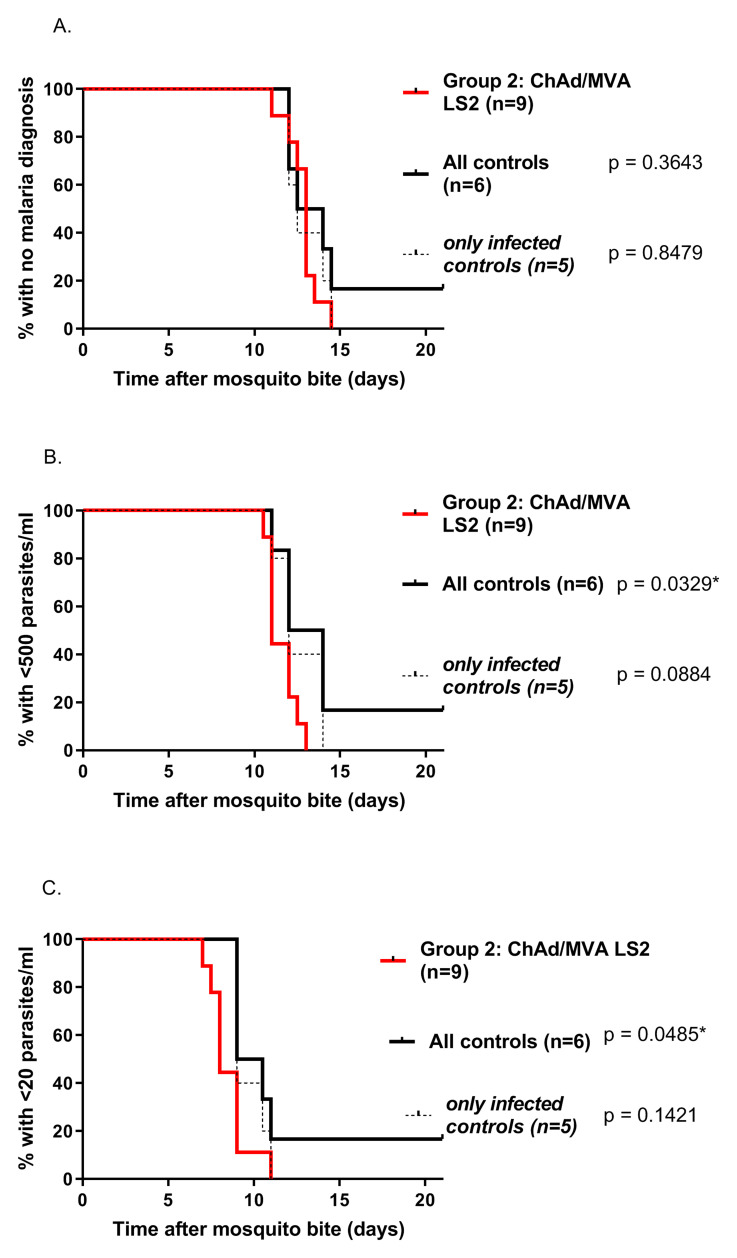
Efficacy of ChAdOx1-MVA LS2 prime boost regimen against *Plasmodium falciparum* 3D7 sporozoite challenge: Kaplan-Meier survival analyses with log-rank comparisons. Group 2 vaccinees (red line) vs controls (black dotted line = all controls). **A**. Primary endpoint. Median time to malaria treatment 13.0d in Group 2 and 13.25d in controls. There was no significant difference in time to diagnosis between vaccinees and controls, p = 0.36. Time to >500 parasites/mL ( **B**) and >20 parasites/mL ( **C**) detected by quantitative polymerase chain reaction (qPCR) showed statistically differences between vaccinees and controls, (p=0.033 and p=0.049 respectively).

**Figure 9.**
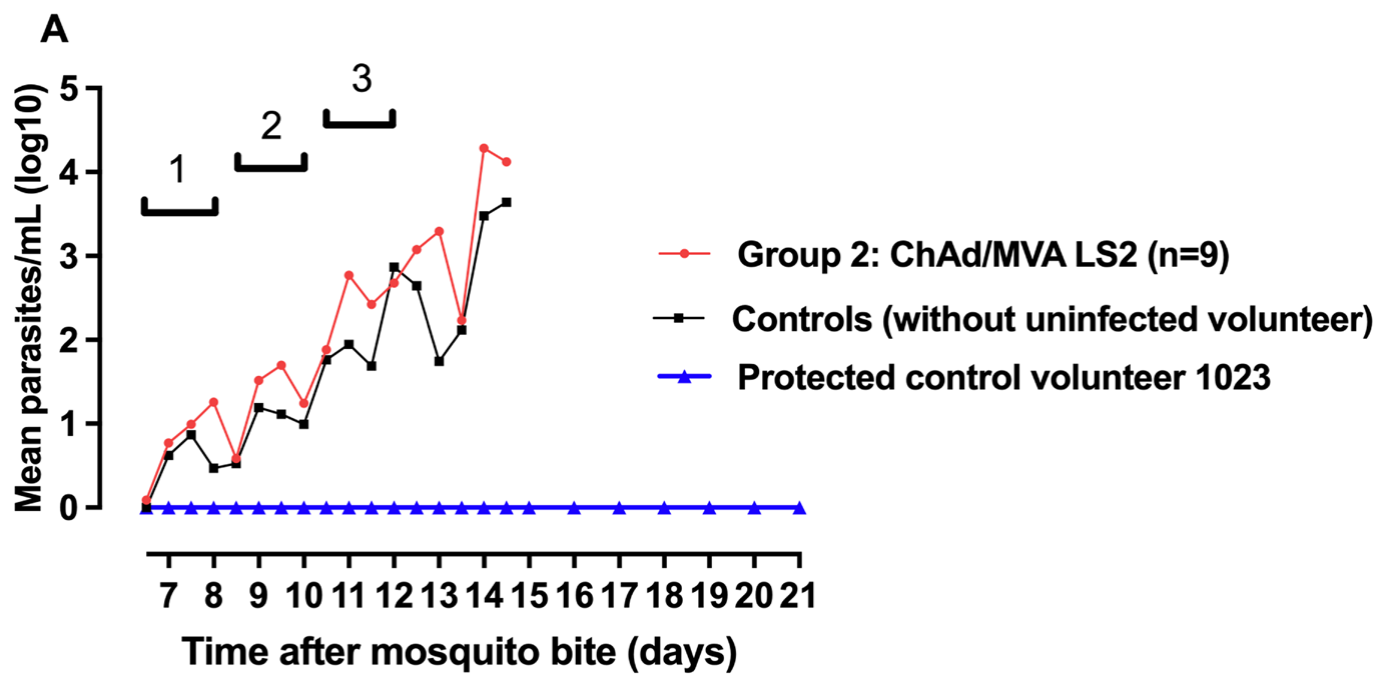
Group mean log-transformed PCR data through post-challenge follow-up. Cycles of parasite replication indicated: cycle 1 – 6.5 – 8.0 days following CHMI; cycle 2 – 8.5 – 10.0 days; cycle 3 – 10.5 – 12.0 days. **A**. Parasite density during the first 2 replication cycles in the erythrocytic stage.

Following mosquito bite challenge, all nine vaccinated volunteers and 5/6 control volunteers were diagnosed with malaria infection during the 21-day follow-up period. Median survival to the primary endpoint (malaria diagnosis) in the vaccine and control arms was 13 (95% CI 12- 13.5 days) and 13.25 (95% CI 12- 14.5 ) days respectively, with no evidence of vaccine induced protection in terms of time to patency ( Figure 8A, p = 0.36, log-rank survival analysis). Survival analysis of time to PCR >500 ( Figure 8B) parasite copies (p)/ml (p = 0.033) and >20 p/ml (p = 0.049; Figure 8C) showed a slightly shorter survival time amongst vaccinees than controls (p=0.088). Mean parasite density following CHMI is shown in Figure 9. There was no significant effect of the vaccine on the liver-emerging parasite burden as defined by mean parasite density 7.5 days after CHMI (p = 0.42 vs all controls; p = 0.72 vs infected controls).

## Discussion

This Phase I/IIa clinical trial demonstrates the safety in a first-in-human administration of a novel *P. falciparum* dual antigenic insert in viral vectors. Testing at the proposed optimum dose 2.5 x 10 ^10^ v.p. was comparable to the previously reported influenza insert. Preliminary comparisons against the leading liver-stage malaria vaccines ChAd63/MVA ME-TRAP did reveal a statistically significant higher occurrence of AEs with LS2. This is based on small numbers of recipients at this stage limiting meaningful comparison and overall ChAdOx1/MVA LS2 was within expected safety limits.

ChAdOx1/MVA LS2 however did not exhibit any evidence of biological protection against malaria using sensitive PCR analysis of parasite replication following release from the liver. Time to infection and parasite density was similar between vaccinees and infected controls. Failure for unvaccinated / naïve participants to develop malaria following five infectious bites in CHMI studies is predicted to be exceedingly rare, approaching 0% ^46^. Though such occurrences could attenuate calculations of efficacy of vaccinated groups by gross numbers infected, given that all vaccinees developed patent malaria, there is minimal impact of this uninfected control participant in this study.

This efficacy result contrasts with the high pre-clinical efficacy predicted by single antigen vaccination with LSA1 and LSAP2 in mice subsequently challenged with transgenic *P. berghei* parasites expressing the relevant *P. falciparum* antigen ^25^. The failure to translate this high efficacy with LS2 does not appear to be accounted for by insufficient magnitude of antigen-specific CD8 ^+^ T cell responses detected by ELISpot. Depletion studies in the mouse model suggest antigen-specific CD8 ^+^ T cells are necessary for protection, particularly for LSAP2 though less convincingly for LSA1 ^25^. Previous vaccines containing LSA1 had failed due to sub-optimal immunogenicity, either because of immune interference from other antigens ^29^, or favoring of antibody induction in the case of LSA-NRC protein in adjuvant ^28^. In our study reported here however, vaccine antigen specific CD8 ^+^ T cell responses at C-1 were both comparable to those seen in a recent study with ChAd63/MVA ME-TRAP and superior to ChAd63/MVA CSP ^19^. Flow cytometric analysis for CD107a also gives good evidence of CTL effector induction by LS2, the presumed mechanism of viral vector mediated protection against liver-stage malaria ^16, 47^.

One likely conclusion therefore is that LSA1 and LSAP2 themselves are not appropriate targets for CTL elimination of *P. falciparum-*infected hepatocytes. This is despite broader association between responses to these antigens (particularly LSA1) and immunity, in the field ^48, 49^ and irradiated sporozoite vaccinated volunteers ^50^ in prior studies. One possible explanation for the lack of protective efficacy is that these antigens are insufficiently presented on the hepatocyte surface when expressed under their natural promoter, compared to the ***UIS4*** promoter in the *P. berghei* model. This poses interesting questions about the fate of LSA1 and LSAP2 within the parasitophorous vacuole, both of which are abundantly expressed though their exact function remains unclear ^26, 27, 51^.

The role for CD8 ^+^ dependent protection in malaria has been highlighted across a range of models including natural infection ^7– 9^. The encouraging immunogenicity of prime-boost vaccination with ChAdOx1/MVA LS2 is informative for future development of vaccines targeting cell-mediated protection, both for malaria and wider contexts. Encoding the antigenic insert adjacent to the short transmembrane domain (TMD) of shark invariant chain showed positive adjuvant effect in the pre-clinical testing and although no comparison of antigens with and without the molecular adjuvant were made in this study. Similarly, expression of vaccine antigen under the F11 promoter within MVA may also be desirable for strong cellular immunogenicity in future vaccine constructs. Notably, the prime boost interval used in this study was short, adding to evidence–as previously indicated in Ebola vaccine trials ^40^–which suggest no adverse impact of shortening the interval between priming and boosting doses.

Viral vector platforms remain an attractive option for the induction of strong CD8 ^+^ T cell responses against liver stage antigens which are crucial for protection in a range of models. Identifying alternative second-generation liver-stage antigens patently expressed on the hepatocyte for clearance by such cells remains a key priority to achieve deployable efficacy for T cell-inducing vaccines against malaria.

## Consent

As part of collecting informed consent, all interested participants screening for the trial were informed that results of this research study may be presented at scientific meetings, conferences or published in a scientific medical journal. All volunteers were informed that they would not be identified in any report or publication.

## Data Availability

Oxford University Research Archive data repository ( https://libguides.bodleian.ox.ac.uk/ora) ^52^: Phase I/IIa study to assess the safety, immunogenicity and efficacy of ChAdOx1-MVA vectored vaccines expressing a novel liver-stage malaria dual antigen LS2 by sporozoite challenge in malaria-naïve adults The project contains the following underlying data: ICS Antibody Information (DOI: 10.5287/ora-eroz5aayp ( http://dx.doi.org/10.5287/ora-eroz5aayp)) ^53^ Safety Data (DOI: 10.5287/ora-x5zyr4mdo ( http://dx.doi.org/10.5287/ora-x5zyr4mdo)) ^54^ Peptide Pools (DOI: 10.5287/ora-gak4agdn9 ( http://dx.doi.org/10.5287/ora-gak4agdn9)) ^55^ qPCR data (DOI: 10.5287/ora-4rapn1kyx ( http://dx.doi.org/10.5287/ora-4rapn1kyx)) ^56^ TREND Checklist (DOI: 10.5287/ora-orkrmjy7r ( http://dx.doi.org/10.5287/ora-orkrmjy7r)) ^57^. Data are available under the terms of the Creative Commons Attribution 4.0 International license (CC-BY 4.0). The data have been de-identified prior to uploading according to the Safe Harbour method.
