# Supplementary material for: Phase I/IIa study to assess the safety, immunogenicity and efficacy of ChAdOx1-MVA vectored vaccines expressing a novel liver-stage malaria dual antigen LS2 by sporozoite challenge in malaria-naïve adults
Source: Wellcome Open Res. 2026 Apr 6;9:734. Originally published 2024 Dec 24. [Version 2] doi: 10.12688/wellcomeopenres.22900.2 (PMC13066805; doi:10.12688/wellcomeopenres.22900.2)
Supplement: Peer review history

# Reviewer report: Reviewer response for version 2

**DOI:** 10.21956/wellcomeopenres.28984.r152819

**Published:** 2026-05-09

**Authors:** Brandon Wilder

**Affiliations:** 1 Oregon Health and Science University Foundation, Beaverton, Oregon, USA

The manuscript is an well-written report on an essential clinical trial advancing a promising malaria liver stage antigen from preclinical to clinical trials. Despite negative results, this reveals important limitations and considerations on the preclinical selection of liver stage antigens. The data collection is complete, but could use some additional interpretation and clarifications before indexing.

 Major points

1. The interpretation of the peptide mapping data should be more robust. Given there is HLA-typing data available this should be incorporated at least in context to the volunteers with responses that map to the same peptides. In addition, data on the proportion of volunteers responding to *any *pool or *any *peptide (above a described positivity cutoff) should be reported. As presented, it is difficult to tell if volunteers 015 and 022 respond to any individual peptides.  
2. The limitations on the antigens and preclinical mouse model in the Discussion is appreciated. However, the last paragraph does not account for another obvious possible shortcoming: that the ChAd-MVA platform as used is not capable of eliciting CD8+ T cells that can target the infected hepatocyte. Mouse and NHP studies have strongly indicated that tissue-resident immunity is key, which typically requires tissue-specific antigen expression/exposure. Given that in the mouse model the transgene-encoded proteins appear to localize to the PVM, this leaves open the possibility that the hypothesis about UIS4 promoter expression is not solely to blame for the lack of prediction in mouse models. Thus, the issue of driving tissue-specific responses (and the ability of this platform to do so) is an essential topic to address so that a reader can have more complete context,

Minor points

1. Would the authors expect a boosting of T cell responses following challenge?
2. A bit more discussion on the safety/efficacy implications of SHIC1 responses in HLA*A02 volunteers is needed.
3. In the results, results are introduced by “Most strikingly” or as an “unexpected signal” without context as to why they are striking or unexpected. Generally, such modifiers and their context should be left for the Discussion unless put in biological context. For the Shark TIM/Ii findings, can the authors include a reference to use of this adjuvant in other clinical trials where no signal was observed?
4. Figure 7B needs to have CD107a on the y-axis. As for ELISpot, the authors should report the proportion of participants which responded to one or either antigen.
5. The results on liver-burden are appreciated, and could be substantiated by clarifying that time to patency was not different between infected controls and infected vaccinees.
6. The assay-focused presentation is completely fine, but to address another reviewers concern the efficacy data could be moved to Figure 1 with the remaining immunogenicity data an exploration of why the vaccine had no efficacy (essentially ruling out lack of immunogenicity).

Is the work clearly and accurately presented and does it cite the current literature?

Yes

If applicable, is the statistical analysis and its interpretation appropriate?

Yes

Are all the source data underlying the results available to ensure full reproducibility?

Yes

Is the study design appropriate and is the work technically sound?

Yes

Are the conclusions drawn adequately supported by the results?

Partly

Are sufficient details of methods and analysis provided to allow replication by others?

Yes

Reviewer Expertise:

Malaria immunology and vaccine development.

I confirm that I have read this submission and believe that I have an appropriate level of expertise to confirm that it is of an acceptable scientific standard, however I have significant reservations, as outlined above.

# Reviewer report: Reviewer response for version 2

**DOI:** 10.21956/wellcomeopenres.28984.r152824

**Published:** 2026-04-20

**Authors:** Ludoviko Zirimenya

**Affiliations:** 1 Department of Clinical Research, London School of Hygiene & Tropical Medicine, Keppel Street, London, UK

Thank you for the opportunity to review this manuscript describing an open-label, dose-escalation, first-in-human phase I/IIa trial in healthy malaria-naïve adults aged 18–45 years. The study aimed to assess the safety, immunogenicity, and preliminary efficacy of a heterologous prime–boost vaccination regimen consisting of ChAdOx1 LS2 followed by MVA LS2.

 The trial included a small lead-in group (group 1; n = 3) who received a reduced dose of ChAdOx1 LS2 (5 × 10⁹ viral particles) for initial safety assessment, followed by a heterologous prime–boost group (group 2; n = 10) receiving ChAdOx1 LS2 (2.5 × 10¹⁰ viral particles) and MVA LS2 (2 × 10⁸ plaque-forming units). Participants in group 2, together with six unvaccinated controls, underwent controlled human malaria infection (CHMI) via mosquito bite. Please find my comments below:

 1.    Allocation and oversight: It is stated that allocation to study groups was undertaken prior to enrolment based on participant preference. Please clarify whether data from the lead-in group (group 1), which received the low dose, were reviewed by an independent data and safety monitoring board (DSMB) before progression to the higher-dose group (group 2). In addition, the role and oversight functions of the DSMB in this trial should be more clearly described.

 2.    Sample size justification: The sample size justification based solely on practical constraints is insufficient. The authors should provide a more quantitative rationale, such as expected effect sizes, precision considerations, or reference to prior controlled human malaria infection (CHMI) studies. If formal power calculations were not undertaken, the implications for interpreting efficacy outcomes should be explicitly acknowledged. 

 3.    Safety assessment: The methods section does not clearly describe how participant safety was assessed. Please specify whether safety evaluations included clinical observation (e.g., vital signs), solicited and unsolicited adverse events, and laboratory assessments (e.g., biochemistry and haematology), and how these were defined and measured. 

 4.    Outcome measures: The manuscript currently presents study objectives rather than clearly defined primary and secondary outcome measures. These should be explicitly stated. In addition, the distinction between Phase 1 (safety/tolerability) and Phase 2a (efficacy in the CHMI model) outcomes should be clarified. 

 5.    Methods vs supplementary material: The methods section includes extensive detail on immunological assays (ELISpot, epitope mapping, flow cytometry) and qPCR. While valuable, much of this information exceeds what is required for interpretation in the main text and would be more appropriately presented in the supplementary appendix, with a concise summary retained in the manuscript. 

 6.    CONSORT flow diagram: In Figure 2 (CONSORT diagram), it would be helpful to clearly indicate the number of participants included in each analysis. In addition, It is noted that 10 participants were enrolled in group 2, but only 9 appeared later as followed-up in the flow diagram. Please clarify what happened to the missing participant. 

 7.    Results structure: The Results section is currently structured by immunological assay, resulting in a method-driven rather than outcome-driven presentation. Consider restructuring the Results to reflect key study endpoints (e.g., safety, efficacy, and immunogenicity), with assay results presented within these domains. In addition, clearer separation of Phase 1 and Phase 2a findings would improve interpretability. 

 8.    Discussion: The Discussion would benefit from a more comprehensive and balanced appraisal of the study’s strengths and limitations. In particular, the authors should address the implications of the small sample size, potential sources of bias (including non-random allocation), and the limited generalisability of findings to malaria-endemic populations.

Is the work clearly and accurately presented and does it cite the current literature?

Yes

If applicable, is the statistical analysis and its interpretation appropriate?

Partly

Are all the source data underlying the results available to ensure full reproducibility?

Yes

Is the study design appropriate and is the work technically sound?

Yes

Are the conclusions drawn adequately supported by the results?

Partly

Are sufficient details of methods and analysis provided to allow replication by others?

Partly

Reviewer Expertise:

Vaccine epidemiology, immuno-epidemiology, and clinical trials of infectious disease interventions

I confirm that I have read this submission and believe that I have an appropriate level of expertise to confirm that it is of an acceptable scientific standard, however I have significant reservations, as outlined above.

# Reviewer report: Reviewer response for version 2

**DOI:** 10.21956/wellcomeopenres.28984.r152696

**Published:** 2026-04-09

**Authors:** Helena Nunes Cabaço

**Affiliations:** 1 Gulbenkian Institute for Molecular Medicine, Lisbon, Portugal

The authors have addressed all comments raised during the review process, and the revised manuscript is suitable for indexing.

Is the work clearly and accurately presented and does it cite the current literature?

Yes

If applicable, is the statistical analysis and its interpretation appropriate?

Yes

Are all the source data underlying the results available to ensure full reproducibility?

Yes

Is the study design appropriate and is the work technically sound?

Yes

Are the conclusions drawn adequately supported by the results?

Yes

Are sufficient details of methods and analysis provided to allow replication by others?

Yes

Reviewer Expertise:

Immunology, Malaria Vaccines

I confirm that I have read this submission and believe that I have an appropriate level of expertise to confirm that it is of an acceptable scientific standard.

# Reviewer report: Reviewer response for version 1

**DOI:** 10.21956/wellcomeopenres.25216.r117060

**Published:** 2025-03-28

**Authors:** Helena Nunes Cabaço

**Affiliations:** 1 Gulbenkian Institute for Molecular Medicine, Lisbon, Portugal

In this article the authors describe a Phase I/IIa study where they assessed the safety and efficacy of ChAdOx1-MVA vectored vaccines, based on a liver stage dual antigen (LS2) strategy. The cellular responses elicited by vaccination were thoroughly studied by ELISPOT and ICS and shown to be high against the antigens. However, no protection was observed upon CHMI.

**The manuscript is fluently written and easy to follow, but some aspects need revision or improvement, as listed below:**

1. In the Results section of the abstract is the word “pertinent” necessary or appropriate?
2. In the Plain Language Summary it’s written “malaria life cycle” – it should be “malaria parasite life cycle”.
3. In the introduction the importance of maintaining durable antibody-mediated responses is mentioned, yet no data on the humoral responses to the vaccine candidates is presented. Could this data be added?
4. In the ICS description it’s mentioned that “PBMCs were permeabilized, then stained intracellularly” but the method or commercial kit used for permeabilization is not mentioned, please add it.
5. In the ICS description there is reference to Table 8 of the extended data, but this is currently Table 1. Also, please include the dilutions of the antibodies and not the volumes.
6. The division of Group 2 in a 2-week vs a 4-week prime-boost schedule only appears in the Results, not in the Methods, where it should be mentioned.
7. Given the complexity of different schedules of prime-boost and sample collection, a diagram with this information (maybe in Figure 4) would improve the comprehension of the procedures.
8. In Figure 4 the information is repeated in B and C, just with different representations. Could the representation used in C be used for all the data? Also, please include in the legend the meaning of the abbreviations in the figure that don’t appear anywhere else (Ch and M, for example).
9. In Figure 4D there is data (for TRAP and CS responses) that is from a different clinical trial. It is not clear from the text whether the ELISPOT was performed at the same time with samples from different clinical trials. Also, is there data from the other clinical trials that can be used to compare the results and understand whether they have not been altered by, for example, time since freezing the cells (although the date of the other trial is not mentioned)?
10. Please modify the y axis in the bottom panel of Figure 7 so that there is no overlay in the text.

Is the work clearly and accurately presented and does it cite the current literature?

Yes

If applicable, is the statistical analysis and its interpretation appropriate?

Yes

Are all the source data underlying the results available to ensure full reproducibility?

Yes

Is the study design appropriate and is the work technically sound?

Yes

Are the conclusions drawn adequately supported by the results?

Yes

Are sufficient details of methods and analysis provided to allow replication by others?

Yes

Reviewer Expertise:

Immunology, Malaria Vaccines

I confirm that I have read this submission and believe that I have an appropriate level of expertise to confirm that it is of an acceptable scientific standard, however I have significant reservations, as outlined above.

# Response

**Published:** 2026-03-18

**Authors:** Katie Ewer

**Affiliations:** GSK Global Vaccines for Global Health Institute, Siena, Italy

We thank the reviewer for the helpful feedback and provide a point-by-point response below. In the Results section of the abstract is the word “pertinent” necessary or appropriate?

- The word ‘pertinent’ has been removed.

  In the Plain Language Summary it’s written “malaria life cycle” – it should be “malaria parasite life cycle”.

- The word ‘parasite’ has been added.

  In the introduction the importance of maintaining durable antibody-mediated responses is mentioned, yet no data on the humoral responses to the vaccine candidates is presented. Could this data be added?

- As our primary was to induce cell-mediated immunity, we did not assess humoral responses in this study.

  In the ICS description it’s mentioned that “PBMCs were permeabilized, then stained intracellularly” but the method or commercial kit used for permeabilization is not mentioned, please add it.

- Details of the Cytoperm/Cytofix kit used have been added to methods section.

  In the ICS description there is reference to Table 8 of the extended data, but this is currently Table 1. Also, please include the dilutions of the antibodies and not the volumes.

- ‘Table 8’ corrected to ‘Table 1’ in the text. The table of antibodies includes both dilutions and volumes.

  The division of Group 2 in a 2-week vs a 4-week prime-boost schedule only appears in the Results, not in the Methods, where it should be mentioned.

- This is described in the study design section of the methods, comment added to signpost this.

  Given the complexity of different schedules of prime-boost and sample collection, a diagram with this information (maybe in Figure 4) would improve the comprehension of the procedures.

- We do not have space to add further figures at this stage.

  In Figure 4 the information is repeated in B and C, just with different representations. Could the representation used in C be used for all the data? Also, please include in the legend the meaning of the abbreviations in the figure that don’t appear anywhere else (Ch and M, for example).

- The data is presented differently to give maximum granularity at the key time points but also representing the overall kinetics of the immune response. We added to the figure legend to explain ‘Ch’ and ‘M’.

  In Figure 4D there is data (for TRAP and CS responses) that is from a different clinical trial. It is not clear from the text whether the ELISPOT was performed at the same time with samples from different clinical trials. Also, is there data from the other clinical trials that can be used to compare the results and understand whether they have not been altered by, for example, time since freezing the cells (although the date of the other trial is not mentioned)?

- We have now updated both the legend and the methods to include details of the previous clinical trial. We have also referenced the clinical study publication.

  Please modify the y axis in the bottom panel of Figure 7 so that there is no overlay in the text.

- This has been done.

# Reviewer report: Reviewer response for version 1

**DOI:** 10.21956/wellcomeopenres.25216.r118989

**Published:** 2025-03-19

**Authors:** Anneth Tumbo

**Affiliations:** 1 Ifakara Health Institute, Ifakara, Tanzania

Thank you for the invitation to review the above-mentioned manuscript

 This well written manuscript which evaluates the safety, immunogenicity and efficacy of chadOX01-MVA vectored vaccines expressing liver stage malaria dual antigen LS2 (Liver stage antigen 1 and LSPA) by sporozoite challenge in malaria naïve adults *. *The study found both vaccine regimens (i.e ChadOx1-L2 and prime boost ChadOx1 and MVA) to be well tolerated. The volunteers were not protected following sporozoite challenge despite the observed immunogenicity of the prime boost regimen as measured by IFNg Eli Spot assay.  The methodology is comprehensive, results are clearly written and well presented. The study limitations are also well acknowledged.

**Comments:**

 Have the authors considered profiling humoral immunity to both vaccines regimens? Would be interesting to see the level of antibody responses induced by both regimens?

Is the work clearly and accurately presented and does it cite the current literature?

Yes

If applicable, is the statistical analysis and its interpretation appropriate?

Yes

Are all the source data underlying the results available to ensure full reproducibility?

Yes

Is the study design appropriate and is the work technically sound?

Yes

Are the conclusions drawn adequately supported by the results?

Yes

Are sufficient details of methods and analysis provided to allow replication by others?

Yes

Reviewer Expertise:

Immuology, Vaccinology, Infectious diseases, Host-parasite interactions

I confirm that I have read this submission and believe that I have an appropriate level of expertise to confirm that it is of an acceptable scientific standard.

# Response

**Published:** 2026-03-18

**Authors:** Katie Ewer

**Affiliations:** GSK Global Vaccines for Global Health Institute, Siena, Italy

We thank the reviewer for their positive feedback. Regarding the comment on further antibody profiling, in light of the lack of protection afforded by the vaccine, we did not undertake futher immunological studies.
